# Supplementary material for: Daily association between perceived control and resolution of daily stressors strengthens across a decade of adulthood
Source: Commun Psychol. 2025 Aug 27;3:130. doi: 10.1038/s44271-025-00313-7 (PMC12390833; doi:10.1038/s44271-025-00313-7)
Supplement: Supplementary file 2 — SupplementaryTables [file 44271_2025_313_MOESM2_ESM.pdf]

Supplemental Material: Supplemental Tables

Supplementary Table 1. Logistic Generalized MLMs for Control Associated with Resolution on Days with One Stressor Reported

	Model 1: Main Effects	Model 2: Moderation by Age Differences	Model 3: Longitudinal Moderation
Parameter	OR [95% CI]	OR [95% CI]	OR [95% CI]
Fixed Effects			
Day	1.01 [0.99, 1.04]	1.01 [0.99, 1.04]	1.01 [0.98, 1.04]
Wave	1.06 [0.92, 1.23]	1.06 [0.92, 1.23]	0.88 [0.62, 1.24]
Women	0.94 [0.81, 1.10]	0.94 [0.81, 1.10]	0.94 [0.81, 1.10]
Race	1.02 [0.81, 1.28]	1.02 [0.81, 1.28]	1.02 [0.81, 1.28]
College	0.67 [0.56, 0.80]***	0.67 [0.57, 0.81]***	0.68 [0.57, 0.81]***
Age at Baseline	0.999 [0.99, 1.01]	0.996 [0.98, 1.01]	0.999 [0.98, 1.01]
WP Stressor Control	1.64 [1.53, 1.75]***	1.65 [1.54, 1.77]***	1.53 [1.41, 1.66]***
WP Stressor Control X Age at Baseline	-	1.003 [0.99, 1.01]	-
WP Stressor Control X Wave	-	-	1.23 [1.06, 1.43]**
BP Stressor Control	1.93 [1.74, 2.14]***	1.93 [1.74, 2.14]***	1.85 [1.64, 2.08]***
BP Stressor Control X Age at Baseline	-	1.002 [0.99, 1.01]	-
BP Stressor Control X Wave	-	-	1.19 [0.94, 1.50]
Level-3 Random Effects			
Intercept Estimate (SE)	0.26 (0.11)	0.26 (0.11)	0.28 (0.11)
Wave Estimate (SE)	-	-	0.19 (0.23)
Level-2 Random Effect			
Intercept Estimate (SE)	0.47 (0.13)	0.47 (0.13)	0.39 (0.16)
-2LL	7047.92	7046.74	7038.11

N=1,712 participants, 5,798 observations. WP=within-person. BP=between-person. Age at Baseline = centered at sample average age at wave 2 (58 years). WP Stressor Control = within-person deviation scores for stressor control. BP Stressor Control = person-mean values for stressor control. We removed the random effect for wave at Level-3 for Model 1 and Model 2 due to estimated G matrix not positive definite. Estimates and standard errors (SE) represent parameter estimates and odds ratio (OR) estimates reflect exponentiated estimates with 95% confidence intervals (CI). †$p<.10$. *$p<.05$. **$p<.01$. ***$p<.001$.

Supplementary Table 2.
Adjusting for Severity in Logistic Generalized MLMs for Control Associated with Resolution of Daily Stressors

	Model 1: Main Effects	Model 2: Moderation by Age Differences	Model 3: Longitudinal Moderation
Parameter	OR [95% CI]	OR [95% CI]	OR [95% CI]
Fixed Effects			
Day	1.02 [1.00, 1.05]†	1.02 [1.00, 1.05]†	1.02 [1.00, 1.05]†
Number of Stressors	2.13 [1.89, 2.40]***	2.13 [1.89, 2.40]***	2.12 [1.88, 2.39]***
Stressor Severity	0.64 [0.58, 0.70]***	0.64 [0.58, 0.70]***	0.64 [0.59, 0.70]***
Wave	1.02 [0.88, 1.18]	1.02 [0.88, 1.18]	0.903 [0.66, 1.24]
Women	1.06 [0.92, 1.23]	1.06 [0.92, 1.23]	1.06 [0.92, 1.23]
Race	1.02 [0.83, 1.26]	1.02 [0.83, 1.26]	1.02 [0.83, 1.26]
College	0.63 [0.53, 0.75]***	0.63 [0.53, 0.75]***	0.64 [0.54, 0.75]***
Age at Baseline	0.998 [0.99, 1.01]	0.995 [0.98, 1.01]	0.998 [0.99, 1.00]
WP Stressor Control	1.60 [1.50, 1.70]***	1.61 [0.50, 1.71]***	1.52 [1.41, 1.64]***
WP Stressor Control X Age at Baseline	-	1.002 [1.00, 1.01]	-
WP Stressor Control X Wave	-	-	1.16 [1.01, 1.33]*
BP Stressor Control	1.84 [1.67, 2.04]***	1.85 [1.67, 2.04]***	1.79 [1.61, 2.00]***
BP Stressor Control X Age at Baseline	-	1.003 [1.00, 1.01]	-
BP Stressor Control X Wave	-	-	1.12 [0.91, 1.39]
Level-3 Random Effects			
Intercept Estimate (SE)	0.38 (0.10)	0.38 (0.10)	0.39 (0.10)
Wave Estimate (SE)	0.01 (0.18)	0.002 (0.18)	0.12 (0.19)
Level-2 Random Effect			
Intercept Estimate (SE)	0.41 (0.13)	0.41 (0.13)	0.36 (0.13)
-2LL	8882.05	8881.16	8876.59

Note. N=1,776 participants, 7,787 observations. Stressor severity was an additional appraisal assessed when stressors were reported using the question, "How stressful was this for you?" and response options on a 4-point Likert-type scale (0=*not at all*, 1=*not very*, 2=*somewhat*, 3=*very*). WP=within-person. BP=between-person. Age at Baseline = centered at sample average age at wave 2 (58 years). WP Stressor Control = within-person deviation scores for stressor control. BP Stressor Control = person-mean values for stressor control. Estimates and standard errors (SE) represent parameter estimates and odds ratio (OR) estimates reflect exponentiated estimates with 95% confidence intervals (CI). †p<.10. *p<.05. **p<.01. ***p<.001.

Supplementary Table 3.
Using Education Level as a Covariate in Logistic Generalized MLMs for Control Associated with Resolution of Daily Stressors

	Model 1: Main Effects	Model 2: Moderation by Age Differences	Model 3: Longitudinal Moderation
Parameter	OR [95% CI]	OR [95% CI]	OR [95% CI]
Fixed Effects			
Day	1.01 [0.99, 1.04]	1.01 [0.99, 1.04]	1.01 [0.99, 1.04]
Number of Stressors	2.17 [1.92, 2.44]***	2.17 [1.92, 2.44]***	2.16 [1.92, 2.43]***
Wave	1.11 [0.96, 1.28]	1.11 [0.96, 1.28]	0.89 [0.65, 1.23]
Women	0.96 [0.83, 1.11]	0.96 [0.83, 1.11]	0.96 [0.83, 1.11]
Race	0.94 [0.76, 1.16]	0.94 [0.76, 1.16]	0.94 [0.76, 1.17]
Education Level	0.93 [0.91, 0.96]***	0.93 [0.91, 0.96]***	0.93 [0.91, 0.96]***
Age at Baseline	0.999 [0.99, 1.01]	0.995 [0.98, 1.01]	0.998 [0.99, 1.01]
WP Stressor Control	1.66 [1.56, 1.77]***	1.67 [1.57, 1.78]***	1.56 [1.45, 1.68]***
WP Stressor Control X Age at Baseline	-	1.002 [1.00, 1.01]	-
WP Stressor Control X Wave	-	-	1.21 [1.06, 1.39]**
BP Stressor Control	1.94 [1.75, 2.14]***	1.94 [1.76, 2.15]***	1.85 [1.66, 2.07]***
BP Stressor Control X Age at Baseline	-	1.003 [0.99, 1.01]	-
BP Stressor Control X Wave	-	-	1.21 [0.97, 1.50]†
Level-3 Random Effects			
Intercept Estimate (SE)	0.38 (0.10)	0.38 (0.10)	0.40 (0.10)
Wave Estimate (SE)	0.07 (0.18)	0.06 (0.18)	0.22 (0.20)
Level-2 Random Effect			
Intercept Estimate (SE)	0.41 (0.13)	0.42 (0.13)	0.35 (0.13)
-2LL	8981.66	8980.58	8971.18

N=1,777 participants, 7,786 observations. Education level ranged from 1-12: 1=*no school/some grade school*, 2=*eight grade/junior high school*, 3=*some high school*, 4=*GED*, 5=*graduated from high school*, 6=1-2 *years of college no degree*, 7=*3+ years of college no degree*, 8=*graduate from 2-year college*, *vocational school, or associate's degree*, 9=*graduated from 4-5 year college or bachelor's degree*, 10=*some graduate school*, 11=*master's degree*, 12=*doctoral degree or other professional degree*. WP=within-person. BP=between-person. Age at Baseline = centered at sample average age at wave 2 (58 years). WP Stressor Control = within-person deviation scores for stressor control. BP Stressor Control = person-mean values for stressor control. Estimates and standard errors (SE) represent parameter estimates and odds ratio (OR) estimates reflect exponentiated estimates with 95% confidence intervals (CI). †p<.10. *p<.05. **p<.01. ***p<.001.

Supplementary Table 4. Within- and Between-Person Associations Among Different Types of Stressor Control and Resolution

	Arguments	Avoided Arguments	Work Stressors	Home Stressors	Network Stressors
Parameter	OR [95% CI]	OR [95% CI]	OR [95% CI]	OR [95% CI]	OR [95% CI]
Fixed Effects					
Day	1.0003 [0.95, 1.05]	1.01 [0.96, 1.05]	1.03 [0.97, 1.08]	1.02 [0.97, 1.07]	1.05 [0.99 – 1.11]
Number of Stressors	0.86 [0.73, 1.004]†	0.79 [0.68, 0.91]**	0.93 [0.79, 1.10]	1.01 [0.86, 1.18]	0.94 [0.79 – 1.12]
Wave	1.33 [0.93, 1.90]	1.26 [0.97, 1.65]†	1.46 [1.02, 2.11]*	1.07 [0.82, 1.40]	0.78 [0.57 – 1.06]
Women	1.28 [0.98, 1.67]†	0.80 [0.63, 1.02]†	1.24 [0.94, 1.63]	1.13 [0.88, 1.45]	0.82 [0.61 – 1.10]
Race	0.92 [0.63, 1.34]	0.93 [0.67, 1.30]	0.88 [0.55, 1.39]	1.03 [0.70, 1.53]	1.23 [0.80 – 1.91]
College	0.72 [0.53, 0.99]*	0.56 [0.42, 0.74]***	0.70 [0.49, 1.01]†	0.68 [0.50, 0.94]*	0.63 [0.45 – 0.88]**
Age at Baseline	1.01 [1.003, 1.03]*	1.01 [1.002, 1.02]*	0.99 [0.98, 1.01]	1.001 [0.99, 1.01]	0.997 [0.99 – 1.01]
WP Stressor Control	1.56 [1.30, 1.86]***	1.51 [1.34, 1.71]***	1.52 [1.28, 1.79]***	1.42 [1.23, 1.65]***	1.49 [1.09 – 2.03]*
BP Stressor Control	1.93 [1.65, 2.26]***	1.65 [1.44, 1.89]***	1.97 [1.69, 2.30]***	1.80 [1.57, 2.06]***	1.38 [1.17 – 1.63]***
Level-3 Random Effects					
Intercept Estimate (SE)	0.10 (0.34)	0.75 (0.26)	0.43 (0.19)	0.17 (0.26)	0.06 (0.42)
Wave Estimate (SE)	0.59 (0.55)	0.12 (0.49)	1.26 (0.65)	0.25 (0.40)	–
Level-2 Random Effect					–
Intercept Estimate (SE)	0.46 (0.42)	0.56 (0.35)	–	0.29 (0.32)	0.18 (0.44)
-2LL	2025.56	3426.73	1851.20	2139.18	1347.24

Note. Arguments: N=911 participants, 1,724 observations. Avoided Arguments: N=1,244 participants, 2,889 observations. Work Stressors: N=757 participants, 1,554 observations. Home Stressors: N=895 participants, 1,659 observations Network Stressors: N=702 participants, 1,028 observations. Age at Baseline = centered at sample average age at wave 2 (58 years). WP Stressor Control = within-person deviation scores for each type of stressor control. BP Stressor Control = person-mean values for each type of stressor control. Estimates and standard errors represent parameter estimates and odds ratio (OR) estimates reflect exponentiated estimates with 95% confidence intervals (CI). We reduced the Work Stressors model to a 2-level model due to estimated G matrix not positive definite. We removed the random effect for wave at Level-3 for the network stressors model due to estimated G matrix not positive definite. Estimates and standard errors (SE) represent parameter estimates and odds ratio (OR) estimates reflect exponentiated estimates with 95% confidence intervals (CI). †p<.10. *p<.05. **p<.01. ***p<.001.

Supplementary Table 5. Descriptive Statistics for Resolution Across Different Types of Stressors

	Wave 2		Wave 3	
Variable	%	Range	%	Range
Any stressor resolution (%)	.69	0 – 1	.68	0 – 1
Argument resolution (%)	.69	0 – 1	.69	0 – 1
Avoided argument resolution (%)	.68	0 – 1	.69	0 – 1
Work stressor resolution (%)	.67	0 – 1	.67	0 – 1
Home stressor resolution (%)	.57	0 – 1	.57	0 – 1
Network stressor resolution (%)	.44	0 – 1	.41	0 – 1

Note. % = Percentage of days when at least one stressor was resolved across stressor days.
